# Metformin and Autoimmunity: A “New Deal” of an Old Drug

**DOI:** 10.3389/fimmu.2018.01236

**Published:** 2018-06-04

**Authors:** Francesco Ursini, Emilio Russo, Gianluca Pellino, Salvatore D’Angelo, Agostino Chiaravalloti, Giovambattista De Sarro, Roberto Manfredini, Roberto De Giorgio

**Affiliations:** ^1^Department of Health Sciences, University of Catanzaro “Magna Graecia”, Catanzaro, Italy; ^2^Colorectal Unit, Hospital Universitario y Politécnico La Fe, Valencia, Spain; ^3^Department of Medical, Surgical, Neurological, Metabolic and Ageing Sciences, Università della Campania “Luigi Vanvitelli”, Naples, Italy; ^4^Rheumatology Institute of Lucania (IReL) – Rheumatology Department of Lucania, “San Carlo” Hospital of Potenza and “Madonna delle Grazie” Hospital of Matera, Potenza, Italy; ^5^Basilicata Ricerca Biomedica (BRB) Foundation, Potenza, Italy; ^6^Department of Biomedicine and Prevention, University Tor Vergata, Rome, Italy; ^7^Department of Nuclear Medicine, IRCCS Neuromed, Pozzilli, Italy; ^8^Department of Medical Sciences, Clinica Medica Unit, University of Ferrara, Ferrara, Italy

**Keywords:** metformin, autoimmunity, autoimmune diseases, T cell, B cell, macrophage, neutrophil, fibroblast

## Abstract

Metformin (*dimethyl biguanide*) is a synthetic derivative of guanidine, isolated from the extracts of *Galega officinalis*, a plant with a prominent antidiabetic effect. Since its discovery more than 50 years ago, metformin represents a worldwide milestone in treatment of patients with type 2 diabetes (T2D). Recent evidence in humans indicates novel pleiotropic actions of metformin which span from its consolidated role in T2D management up to various regulatory properties, including cardio- and nephro-protection, as well as antiproliferative, antifibrotic, and antioxidant effects. These findings, together with ground-breaking studies demonstrating its ability to prolong healthspan and lifespan in mice, provided the basis for defining metformin as a potential *antiaging* molecule. Moreover, emerging *in vivo* and *in vitro* evidence support the novel hypothesis that metformin can exhibit immune-modulatory features. Studies suggest that metformin interferes with key immunopathological mechanisms involved in systemic autoimmune diseases, such as the T helper 17/regulatory T cell balance, germinal centers formation, autoantibodies production, macrophage polarization, cytokine synthesis, neutrophil extracellular traps release, and bone or extracellular matrix remodeling. These effects may represent a powerful contributor to antiaging and anticancer properties exerted by metformin and, from another standpoint, may open the way to assess whether metformin can be a candidate molecule for clinical trials involving patients with immune-mediated diseases. In this article, we will review the available preclinical and clinical evidence regarding the effect of metformin on individual cells of the immune system, with emphasis on immunological mechanisms related to the development and maintenance of autoimmunity and its potential relevance in treatment of autoimmune diseases.

## Introduction

Since its discovery more than 50 years ago, metformin represents a worldwide milestone in treatment of patients with type 2 diabetes (T2D). Metformin (*dimethyl biguanide*) is a synthetic derivative of guanidine, isolated from the extracts of *Galega officinalis*, a plant with a prominent antidiabetic effect (1). Indeed, metformin lowers both fasting and post-prandial glucose levels by inhibiting hepatic glucose production, reducing intestinal glucose absorption, and improving glucose uptake and utilization by peripheral tissues. However, the exact pharmacodynamic properties have been elusive for many years still remaining a matter of debate (2). Recent evidence in humans indicates novel pleiotropic actions of metformin which span from its consolidated role in T2D management up to various regulatory properties, including cardio- and nephro-protection, as well as antiproliferative, antifibrotic, and antioxidant effects (3). These findings, together with ground-breaking studies demonstrating its ability to prolong healthspan and lifespan in mice (4), provided the basis for defining metformin as a potential *antiaging* molecule (5). This fascinating hypothesis is currently being tested through the ongoing Targeting Aging with Metformin study, a placebo-controlled trial (5) aimed at investigating the potential role of metformin in delaying the onset of aging-associated diseases.

Notably, emerging *in vivo* and *in vitro* evidences suggest that metformin can exhibit immune-modulatory features (6, 7). These effects may represent a powerful contributor to antiaging and anticancer properties exerted by metformin.

In addition to recent evaluation of the metabolic and immunological roles of metformin (7), this article will appraise current preclinical and clinical data regarding the effect of metformin on individual cells of the immune system. Furthermore, the immunological mechanisms exerted by metformin will be explored as well as its potential relevance in the treatment of autoimmune diseases.

## Pharmacokinetics and Mechanism of Action of Metformin

### Pharmacokinetics

Upon oral administration in humans (typical therapeutic doses range from 1,000 to 3,000 mg/day), approximately 70% of metformin is absorbed from the small intestine (8), with the remaining component passing into the colon before being excreted in feces (9). The intestinal absorption is primarily mediated by the plasma membrane monoamine transporter, which is expressed on the luminal side of enterocytes; however, the organic cation transporters 1 and 3 may also contribute (10). Following absorption, metformin is mainly distributed in the intestine, liver, and kidneys and finally excreted unchanged in urine by means of active tubular secretion, with half-life of ~5 h (8).

### Mechanism of Action

Consistent data show that mitochondria are the main subcellular targets of metformin. Indeed, the drug accumulates selectively in mitochondria reaching up to 1,000-fold higher concentrations than those observed in the extracellular medium (11). Recent studies demonstrate that metformin transiently inhibits NADH:ubiquinone oxidoreductase (also referred to as “Complex I” of the mitochondrial electron transport chain), an entry enzyme of oxidative phosphorylation located in the inner mitochondrial membrane. This inhibitory effect evokes a drop in the energetic state of the cell, thereby leading to a reduced ATP production and increased AMP:ATP ratio (2). The resultant metabolic shift leads to the activation of the energy sensor 5′-AMP-activated protein kinase (AMPK). Upon activation, AMPK coordinates different signaling networks in the attempt to restore a physiologic energy balance by switching on ATP-generating catabolic pathways and, at the same time, shutting down ATP-consuming anabolic mechanisms. The most of the well-characterized glucose-lowering effects of metformin relies on AMPK activation, although AMPK-independent mechanisms have been recently disclosed (2).

Downstream consequences of AMPK activation may account for many of the effects of metformin on immune homeostasis. Indeed, immunological functions are associated with specific metabolic pathways and targeting immunocytes. Upon activation, these cells, even those with prominent anti-inflammatory properties, undergo metabolic reprogramming that is essential for disease development and maintenance (12, 13). Pro-inflammatory cell subset, i.e., neutrophils, M1 macrophages, and effector T cells preferentially produce ATP through glycolysis, whereas cells with an anti-inflammatory lineage, i.e., memory and regulatory T cells (Tregs) and M2 macrophages, favor mitochondrial ATP generation (13). In this context, AMPK activation promotes the oxidation of substrates in mitochondria, thereby limiting the glycolytic capacity of cells (14).

The mammalian target of rapamycin (mTOR) is a downstream target of AMPK involved in regulating energy homeostasis by modulating cellular processes, such as protein synthesis and autophagy implicated in cell proliferation and tumorigenesis in cancer (15). With regard to immune function, mTOR is crucial for fine tuning T cells activation and differentiation (16) through its interaction with the signal transducer and activator of transcription (STAT) pathway (17). Metformin has been demonstrated to downregulate mTOR signaling through either AMPK-dependent (18) or -independent mechanisms (19, 20). Moreover, metformin can regulate other pathways relevant to autoimmunity, including the nuclear factor kappaB (NF-kB) (21) and mitogen-activated protein kinase (MAPK)/c-Jun NH2-terminal kinase (JNK) (22).

## Effects of Metformin on Immune Cells Involved in Autoimmune Diseases

**Figure 1:**
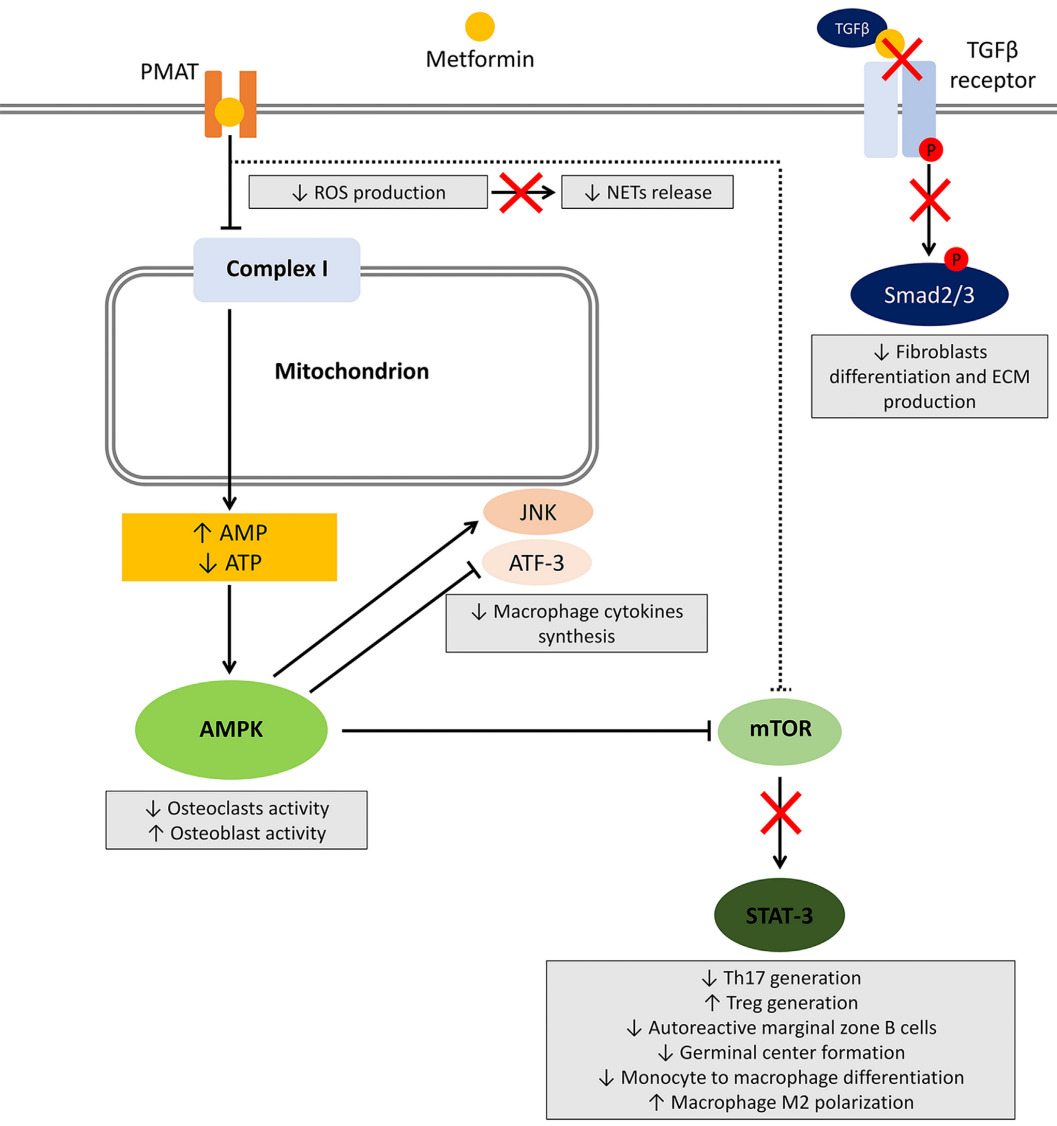
Effects of metformin on immune cells. After entering the cell, metformin transiently inhibits NADH:ubiquinone oxidoreductase (complex I) located in the inner mitochondrial membrane leading to a reduced ATP production and increased AMP:ATP ratio. The resultant metabolic shift stimulates the activation of the energy sensor 5'-AMP-activated protein kinase (AMPK). Among several targets, AMPK activation inhibits mammalian target of rapamycin (mTOR), responsible for most of the effects on the immune system. Other potential mechanisms include AMPK-independent inhibition of mTOR, reduced reactive oxygen species (ROS) production, and block of the TGF-β receptor/Smad interplay. The effects on immune cells are summarized in gray boxes.

Several studies investigated the effect of metformin on different cells involved in the induction and maintenance of autoimmunity and inflammation. Herein, we will review the available evidence regarding the effect of metformin on main cell types and related pathways (summarized in Figure 1).

### Effects of Metformin on T Cells

T cells are key master players in the delicate equilibrium leading to immune tolerance breakdown and autoimmunity (23). The traditional dichotomy between Th1- and Th2-mediated diseases has been overwhelmed in the last years by the identification of novel T cell subsets, including T helper 17 (Th17) and Treg populations. Th17 cells are characterized by the production of the pro-inflammatory cytokine IL-17 and the expression of the transcriptional factor retinoic-acid-receptor-related orphan nuclear receptor gamma (RORγt) and exert pro-inflammatory effects relevant to the pathophysiology of several autoimmune diseases (24). Conversely, Tregs are characterized by the expression of the transcription factor forkhead box P3 and act as negative regulators of immune-mediated inflammation (25). Globally, their balance represents the subtle edge on which autoimmunity develops and maintains chronically (25, 26). T cell metabolic derangement has been largely demonstrated in autoimmune diseases such as systemic lupus erythematosus (SLE), with a shift toward oxidation, mitochondrial abnormalities, activation of mTORC1, and increased glucose flux (27).

In this context, the mTOR pathway—a key downstream molecule of AMPK signaling—and its crosstalk with STAT-mediated signaling, plays a crucial role in regulating of T cell subset differentiation (17, 28). mTOR-deficient T cells fail to differentiate into Th1, Th2, or Th17 effector cells, while maintaining their ability to differentiate into Tregs (29). Specularly, the addition of rapamycin (mTOR inhibitor) to CD25-cell cultures selectively induces the Treg phenotype and function (30). Finally, treatment with mTOR-targeting agents, such as sirolimus (31) or N-acetylcysteine (32), improves disease activity in patients with SLE.

Nath et al. (33) evaluated the effect of oral metformin on experimental autoimmune encephalomyelitis (EAE), a T-cell-mediated mouse model of multiple sclerosis (MS). In this study, treatment with metformin resulted in a slower disease progression, reduced infiltration of inflammatory cells in the central nervous system and expression of inflammatory cytokines IFN-γ, TNF-α, IL-17, IL-1β, and IL-6. Notably, T cells isolated from mice treated with metformin showed reduced expression of IFN-γ and IL-17 along with the two transcription factors T-box transcription factor and RORγt suggestive of Th1 and Th17 differentiation, respectively. A similar effect was obtained when metformin was associated with lovastatin (34). Complementary with the reduction of Th17, metformin has been also demonstrated to increase Tregs in EAE through the inhibition of mTOR pathway (35).

Similarly, Kang et al. evaluated the effect of metformin on collagen antibody-induced arthritis (CAIA), a well-established animal model of rheumatoid arthritis (RA) (36). In CAIA mice, metformin treatment resulted in a significant improvement of arthritis score, with reduced bone destruction, inflammatory cytokines production, and RORγt-expressing T cells associated with the AMPK/mTOR-mediated inhibition of STAT3 signaling.

The beneficial AMPK/mTOR/STAT3-dependent effect of metformin on Th17-mediated inflammation was further confirmed in collagen-induced arthritis (CIA) (37–39), dextran sulfate sodium-induced colitis (reminiscent of human inflammatory bowel disease, IBD) (40), Roquin^san/san^ model of SLE (41), and acute graft-versus-host disease (42).

In addition to the AMPK/mTOR/STAT3 pathway, other T cell pathogenic mechanisms may be targeted by metformin. Several other metabolic pathways regulate the function of T cells in health and diseased conditions (43, 44). Mitochondrial oxidative metabolism has been shown to be the main ATP synthesis pathway in experimental SLE (27, 45). Using the triple congenic B6.Sle1.Sle2.Sle3 lupus-prone mouse model, Yin et al. (46) demonstrated that metformin can restore T cell metabolism and reduce IFN-γ production. Moreover, the simultaneous treatment of mice with metformin and 2-deoxy-d-glucose—a glycolysis inhibitor—significantly reduced anti-nuclear antibodies and anti-double-stranded DNA (dsDNA) antibodies production and improved the severity of associated nephritis. Similar findings were obtained from the same group in B6.lpr mice, another model of spontaneous SLE (47).

Taken together, data from animal models of T cell-mediated autoimmunity strongly support an immune-modulatory effect of metformin with the ability to restore the balance between pathogenic and tolerogenic T cell populations by acting on the AMPK/mTOR/STAT3 and on the normalization of T cell mitochondrial metabolism.

Despite the promising results obtained with experimental models, data in humans are still scarce. Yin et al. (46) demonstrated that CD4^+^ T cells from SLE patients exhibit elevated cellular metabolism when compared with healthy controls, a feature that correlates with T cell activation and subset distribution. Moreover, their excessive IFNγ production was significantly reduced by adding metformin *in vitro*. Furthermore, immune function is known to be impaired in T2D, a feature that explains an increased susceptibility to infection and autoimmune diseases (48). Dworacki et al. (49, 50) showed that T2D is characterized by impaired thymic output, as documented by reduced number of circulating recent thymic emigrants (RTEs) and CD127^+^ CD132^+^ naïve T cells, mainly attributable to the conversion of RTE in terminally differentiated memory cells. Among diabetic patients, however, those treated with metformin had the highest thymic output of a similar magnitude to that observed in nondiabetic subjects (50). IL-17 and IFN-γ levels were higher in patients with poor glucoregulation at baseline (defined as a glycated hemoglobin >7%); nonetheless, a 12-week treatment with metformin could reduce IL-17 in diabetic patients. Finally, in patients with polycystic ovary syndrome (PCOS), the administration of metformin combined with drospirenone/ethinylestradiol has been shown to reduce the frequency of CD4^+^CD28^null^ T cells (51), a subset of CD4^+^ Th1 cells lacking the co-stimulatory receptor CD28 thought to be involved in autoimmune diseases such as RA (52).

Only limited direct evidence of the potential therapeutic effect of metformin in patients with autoimmune diseases are available to date. In a cohort study by Negrotto et al. (53), MS patients with comorbid metabolic syndrome were treated with metformin (850–1,500 mg/day) or pioglitazone resulting in a significant decrease in enlarging T2-weighted and gadolinium-enhanced lesions (as a measure of disease burden) on brain magnetic resonance imaging compared with matched controls. This result was already evident after 6 months of therapy and maintained up to 24 months. Moreover, in peripheral blood mononuclear cell isolated from patients treated with metformin, the authors observed an enhanced AMPK expression, a reduced prevalence of IFN-γ- and IL-17-producing cells, and an increase of Treg cell percentage. In a proof-of-concept trial by Wang et al. (54), 113 SLE patients were randomly assigned to receive add-on (500–1,500 mg/day) metformin or conventional treatment alone. Metformin treatment resulted in a 51% reduction of risk of disease flares and significantly less corticosteroid exposure.

### Effects of Metformin on B Cells

In the network of mutual interactions between immune cells, the interplay between T and B cells represents a crucial component for the development of adaptive immune responses (55). Despite many autoimmune conditions have been considered T cell-driven diseases for a long time, several B cell-dependent mechanisms are emerging in recent years extending beyond their traditional role as autoantibody-producing cells (56, 57).

The stronger support for a potential role of metformin on B cells biology arises from studies investigating its effect on hematological malignancies. Several lines of evidence indicate that aberrant activation of the mTOR pathway is common in both Hodgkin lymphomas and many types of B-cell non-Hodgkin lymphomas (58), thereby contributing to tumorigenesis and cell autophagy during response to anticancer agents. Notably, defects in autophagy mechanisms has recently been proposed to play a role in lymphoma progression, thus implying that autophagy represents a promising target for novel lymphoma therapeutics (59). Notably, mTOR-driven autophagy is also an important mechanism in autoimmune diseases (60, 61).

Shi et al. (62) reported the antitumor action of metformin-mediated AMPK activation in lymphoma. Metformin treatment induced a dose-dependent suppression of lymphoma cells proliferation through negative control of the mTOR pathway. Importantly, lymphoma cells sensitivity to anticancer agents was enhanced by concomitant treatment of metformin *via* induction of autophagy. Furthermore, metformin use has been associated with an improved response rate and progression-free survival in diabetic diffuse large B-cell lymphoma patients (63).

Recent studies suggested that mTOR activity plays a critical role in B cells during autoimmune diseases. Conditional deletion of the mTOR genes in B cells markedly impairs B cell proliferation and germinal center (GC) differentiation (64). Using the Roque^san/san^ model of SLE, Lee et al. (41) demonstrated that oral metformin can attenuate signs of autoimmunity including anti-dsDNA antibodies production and kidney and liver inflammation. This clinical improvement was accompanied by a significant reduction in autoreactive marginal zone B cells and reduced GC formation, the main site of differentiation of B cells in long-lived autoreactive plasma cells. At the molecular level, this was characterized by an increased activation of AMPK with subsequent decrease in phosphorylation of mTOR and STAT3 (41). Conversely, protective antibody response may be even boosted by metformin. Diaz et al. (65) evaluated the antibody response to influenza vaccination in T2D patients’ naïve and on metformin treatment, respectively. The drug enhanced vaccine-specific antibody titers *in vivo* and *in vitro*, and this effect was accompanied by an increase in switched memory B cell and a decrease in late/exhausted B cells, known to impair antibody responses. These mechanisms were associated with an increased AMPK phosphorylation and reduced intrinsic B cell inflammation upon exposure to metformin, as demonstrated by a reduction in TNF-α, miR-155, and miR-16 expression, known to affect the ability of B cells to respond to antigenic stimulation (41).

Among several B-cell subpopulations so far identified (66), the innate-like B-1a cells represent a distinct subpopulation that can significantly contribute to generate circulating IgM natural antibodies, mucosal immunity, and immunoregulation (67, 68). Furthermore, two distinct phenotypes have been identified in B-1a cells. These are distinguished by the relative expression of the plasma cell alloantigen 1 (PC1, also known as ENPP1—ectonucleotide pyrophosphatase/phosphodiesterase 1) and are named PC-1^low^ and PC-1^high^, with the latter subsets exhibiting a more pronounced regulatory function (69, 70). The same glycoprotein PC-1 has been demonstrated to have a role in insulin resistance associated with T2D (71). Stefanovic et al. (72) demonstrated an increased PC-1 expression in T2D lymphocytes, which was reverted by a 3-month metformin treatment and combined with a significant improvement of insulin sensitivity. Several lines of evidence support a potential role of B1a cells in the development of autoimmunity (73); thus, the modulation of PC-1 by metformin may represent an additional immune-regulatory mechanism deserving further studies.

### Effects of Metformin on Monocytes/Macrophages

Macrophages represent the main tissue-resident immune cells in many organs providing a quick first-line response against pathogens. Upon activation, macrophages can polarize in two major phenotypes, i.e., the pro-inflammatory “classically” activated (M1) and the “alternatively” activated (M2, further subclassified as M2a, b, or c), mainly associated with resolution of inflammation and tissue repair processes (74, 75). Inflammatory infiltrates in autoimmune diseases are characterized by a predominance of a distinct macrophage lineage: active RA synovial tissue shows abundance of M1 macrophages, whereas in spondyloarthritis patients, the M2 phenotype predominates (76). Consistently with their respective functions, the cytokine portfolio is significantly diversified, with M1 macrophages producing mainly pro-inflammatory cytokines relevant to the pathogenesis of autoimmune diseases (i.e., TNF-α, IL-1, IL-6, IL-12, IL-23, and MCP-1), as opposed to M2 macrophages releasing cytokines with anti-inflammatory properties, e.g., IL-10 and TGF-β (77).

*In vitro*, metformin demonstrated anti-inflammatory properties on macrophages *via* AMP-dependent and -independent mechanisms. In THP-1 acute monocytic leukemia cell line, metformin treatment abrogates phorbol 12-myristate 13-acetate-induced monocyte-to-macrophage differentiation and IL-1β, TNF-α, and MCP-1 production (78). This effect is primarily mediated by AMPK activation resulting in a decrease in JNK1 phosphorylation (influencing inflammatory cytokine synthesis and release) and the negative regulating STAT3 phosphorylation (modulating the effects on differentiation). In primary murine peritoneal macrophages, metformin treatment dose-dependently suppresses lipopolysaccharide (LPS)-induced TNF-α and IL-6 expression. This effect was attributable, at least in part, to the AMPK-dependent ATF-3 induction *via* competition with NF-κB for binding to TNF-α and IL-6 promoters (79). Similarly, Kelly et al. (80) demonstrated that metformin dose-dependently inhibits LPS-induced pro-IL-1β while boosting IL-10 expression in murine bone marrow-derived macrophages. In this case, the effect on pro-IL1β was independent of AMPK activation and associated with reduced reactive oxygen species (ROS) production as a direct consequence of metformin-induced mitochondrial complex I suppression. In high-fat diet (HFD) mice, a classical model of obesity-induced T2D and chronic inflammation, treatment with metformin resulted in reduced serum levels of IL-6 and TNF-α and in the AMPK-mediated modulation of macrophage polarization with a shift toward an anti-inflammatory M2 phenotype (81).

Similarly, in human macrophages, metformin was able to suppress the LPS-induced expression of TNF-α and MCP-1 and ROS production in an AMPK-dependent manner. This effect was associated with a reduced NF-kB and MAPK activity (82). In impaired glucose tolerance patients treated with fenofibrate, addition of metformin for 12 weeks was able to reduce LPS-induced production of TNF-α and IL-6 (83) by peripheral blood monocytes. Likewise, in impaired fasting glucose patients treated with simvastatin, addition of metformin was able to reduce LPS-induced TNF-α, IL-1β, IL-6, IL-8, and MCP-1 (84). Using cultures of human adipose tissue, Bruun et al. (85) demonstrated that metformin reduces the release of MCP-1 by resident macrophages.

Macrophage migration inhibitory factor (MIF) is a pleiotropic cytokine, acting as potent M1-polarizing factor (86, 87). Evidence for a role for MIF in autoimmunity has been provided by studies showing that MIF is expressed at increasing levels in different experimental models of disease. Immunoneutralization or genetic deletion of MIF confers protection from pathologic progression (88–91). In addition, both the circulating levels and the tissue expression of MIF are elevated in patients with autoimmune inflammatory disorders, and high-expression MIF alleles have been associated with more severe end-organ damage in RA (92, 93), SLE (94), and scleroderma (95). As for the macrophage polarization, synovial fluids in RA patients contain high levels of M1 macrophage-derived mediators, along with low levels of M2 macrophage-derived mediators (96). Adoptive transplantation of M2, but not M1, macrophages significantly reduced SLE severity in lymphocyte-derived DNA (ALD-DNA) induced lupus mice (97). Dandona et al. (98) demonstrated that plasma MIF concentrations and MIF mRNA expression in the mononuclear cells are elevated in obese patients, and oral metformin treatment for 6 weeks suppresses MIF levels. Further studies are eagerly awaited to further define the role of MIF in autoimmune diseases.

### Effects of Metformin on Neutrophils

Neutrophils are the most numerous circulating leukocytes in humans, providing a powerful first-line defense against bacterial and fungal pathogens. Despite these cells densely infiltrate various tissues in autoimmune diseases, their exact role was puzzling until the last years (99), when a number of studies demonstrated the involvement of neutrophils in different phases of autoimmune diseases pathogenesis (100).

From a clinical standpoint, the neutrophil count and neutrophil-to-lymphocyte ratio (NLR, normal range: 0.78–3.53) emerged in the last years as an accessible, inexpensive measure of systemic inflammation (101). NLR has been extensively studied as a prognostic marker in cancer patients (102); moreover, it correlates with disease activity in SLE (103), RA (104), MS (105), and IBD (106). In a large cohort of diabetic patients, Cameron et al. (107) demonstrated that treatment with metformin, but not with sulfonylureas, significantly reduces NLR after 8–16 months. Also, metformin has been shown to reduce neutrophil count in young women with PCOS (108) and in girls born small for gestational age with exaggerated adrenarche and precocious pubarche (108), two conditions characterized by a pronounced systemic inflammatory state. Finally, in diabetic patients undergoing endarterectomy for carotid artery stenosis, Eilenberg et al. (109) demonstrated that metformin treatment was associated with reduced expression of neutrophil gelatinase-associated lipocalin—a protein released by activated neutrophils and associated with atherosclerotic plaques vulnerability—thus contributing to lower cerebral embolization events.

More recently, a novel, exciting feature of neutrophils has been disclosed, namely, the production of neutrophil extracellular traps (NETs) (110). NETs are DNA structures released upon chromatin decondensation and spreading in the extracellular space. Several proteins adhere to the DNA scaffold, including histones and components of primary and secondary granules, such as elastase, myeloperoxidase, cathepsin G, lactoferrin, pentraxin 3, gelatinase, proteinase 3, LL37, and peptidoglycan-binding proteins. Exaggerated NETosis is increasingly being recognized as a contributing mechanism in induction and maintenance of autoimmunity and a major source of autoantibodies generation in SLE (111) and RA (112). Furthermore, Wang et al. demonstrated that mitochondrial DNA, in addition to nuclear DNA, can be found in NETs obtained by stimulating neutrophils from SLE patients and anti-mitochondrial DNA antibody response associates with disease activity even better than anti-dsDNA. Moreover, treatment with metformin in SLE patients resulted in a reduction of disease flares and corticosteroid use (54). NETosis can be experimentally induced *in vitro* following exposure of human peripheral blood white cells to high glucose concentration (113); similarly, exaggerated NETosis has been clearly demonstrated in patients with T2D (113, 114), and this process can be restored by treatment with metformin (115). Finally, in patients with pre-diabetes, metformin treatment reduces the concentration of NET components independently from glycemic control (116).

## Effects of Metformin on Other Cells Involved in Autoimmune Diseases

### Effects of Metformin on Fibroblasts

Fibroblasts actively participate in the pathophysiology of several autoimmune conditions (117), but their role is emphasized in systemic sclerosis (SSc)—the prototypical systemic fibrosing disease—in which activated myofibroblasts drive uncontrolled extracellular matrix accumulation in the skin and internal organs. At a subcellular level, TGF-β is the key cytokine regulating fibroblasts aggressive behavior in SSc (118). TGF-β signals through cell-surface serine/threonine kinase receptors to the intracellular Smad proteins, which in turn accumulate in the nucleus to regulate gene expression (119).

Extensive preclinical data suggest a potent antifibrotic effect of metformin. In cardiac fibroblasts, metformin treatment has been shown to inhibit fibrosis and collagen synthesis *via* the TGF-β/Smad3 signaling pathway (120) and to impair the differentiation into myofibroblasts (121). A similar effect was observed in other cellular models, including nasal polyp-derived fibroblasts (122), renal fibroblasts (123), hepatic stellate cells (124), and in fibroblasts harvested from capsular ligaments of ankylosing spondylitis patients (125). In lungs, metformin attenuates gefitinib-induced exacerbation of pulmonary fibrosis by inhibiting the TGF-β/SMAD2/3 signaling pathway (126). Finally, our group demonstrated that metformin can ameliorate skin fibrosis in the bleomycin-induced murine model of scleroderma (127), as indicated by reduced dermal thickness, collagen accumulation, and number of lesional myofibroblasts. The mechanisms leading to metformin-mediated downregulation of the TGF-β axis have been elusive until recently, when an elegant study demonstrated that metformin is able to directly bind to TGF-β, thereby preventing the interaction with its receptor (128).

### Effects of Metformin on Osteoblasts/Osteoclasts

Under physiological conditions, bone homeostasis is maintained through a harmonic balance between bone formation and resorption, orchestrated by osteoblasts and osteoclasts, respectively (129). During RA and other autoimmune diseases, pro-inflammatory cytokines such as IL-1 and TNF-α alter this subtle equilibrium in favor of bone-resorptive mechanisms, ultimately leading to local (bony erosions) (130) and systemic (osteoporosis) consequences (129). AMPK plays an important role in regulating bone turnover by suppressing osteoclasts (131) while concurrently stimulating osteoblasts (132). Consequently, metformin exhibited protective effects in animal models of osteoporosis (133, 134). Furthermore, Son et al. (37) demonstrated that improvement in bone erosion and cartilage destruction in CIA mice following exposure to metformin is associated with reduced osteoclastogenesis *via* an AMPK/mTOR/STAT3-dependent mechanism. In humans, it is well accepted that T2D is associated with an increased risk of osteoporosis (135). While some antidiabetic medications, such as thiazolidinediones, demonstrated clear pro-osteoporotic effects (136), metformin treatment has been associated with reduced risk of fractures in a large, case–control study (137). So far, there are no data on the effect of metformin on bone homeostasis in patients with rheumatic disease.

## Metformin and Gut Microbiota

Growing evidence indicates that the gut microbiota—i.e., the approximately 100 trillion germs located into the gastrointestinal lumen, especially in the distal segments—plays a role in human body homeostasis and health state (138). Alterations in the gut microbiota composition, a condition referred to as *dysbiosis*, has been associated with the development of several autoimmune diseases, including RA (139), SLE (140), MS (139), and Behcet’s disease (141).

Several factors and agents have been shown to interfere or even modulate the microbiota; however, little is known about the effects exerted by metformin on human gut and *vice versa*. Cabreiro et al. (142) investigated the impact of metformin on life cycle of the nematode *Caenorhabditis elegans*. Following on previous studies showing that metformin extended the lifespan of *C. elegans* (143), the authors aimed at investigating the mechanisms underlying these effects and demonstrated that metformin slowed aging of *C. elegans* rather than reducing the risk of death. Furthermore, using *Escherichia coli* co-cultures, they found that metformin inhibits bacterial folate and methionine metabolism, both mechanisms thought to contribute significantly the therapeutic efficacy of metformin. The conclusions support the *C. elegans/E. coli* theory of evolution (144), which implies that the animal or plant, with all associated microorganisms, are considered a “unit” of selection during evolution (“the holobiont”). Under this light, studies aiming at understating the mechanisms mediating the effects of metformin need to be conducted on adequately designed models and will require accurate assessment of the gut microbiota.

Further evidence of an axis connecting the gut microorganisms with metformin mechanism of action is provided by an experimental study in which normal diet or HFD mice were treated with metformin for 6 weeks. Compared to untreated (control) HFD mice, those receiving metformin improved the glycemic profile, and this effect was associated with higher abundance of *Akkermansia muciniphila*, a mucin-degrading bacterium. Furthermore, oral administration of *A. muciniphila* reduced visceral adipose tissue inflammation and enhanced glucose tolerance in HFD mice (145). Similar findings were confirmed at some extent by Lee and Ko (146) who showed that the abundance of *A. muciniphila* (12.44 ± 5.26%) and *Clostridium cocleatum* (0.10 ± 0.09%) significantly increased after metformin treatment in HFD mice. The same authors also addressed the role of *Akkermansia* and other microbiota genera in obese, aged mice treated with metformin (147). HFD mice treated with metformin had significantly increased abundance of *Akkermansia, Bacteroides, Butyricimonas*, and *Parabacteroides*, and this correlated with a reduced expression of inflammatory cytokines (IL-1β and IL-6) in the adipose tissue. It has been suggested that metformin treatment could restore glucose sensing by regulating the expression of small intestinal sodium glucose cotransporter-1 in rats, which is reduced by HFD. Upper small intestine treatment with metformin has been shown to change the microbiota by increasing, at least in part, the abundance of *Lactobacillus* (148). Finally, Wu et al. assessed metformin–microbiota interactions in a gut simulator and confirmed that metformin alters the biological functions of different microbial phyla, including the regulation of genes encoding for metalloproteins or metal transporters (149).

Several clinical studies investigating the relationship between gut microbiota and metformin treatment have been performed in subjects with T2D. Forslund et al. (150) analyzed 784 human metagenomes and observed a shift in the microbiota during metformin treatment with a depletion of butyrate-producing taxa. They proposed the potential role of a microbial influence on the effects of metformin, which could be related with short-chain fatty acids production (151). In keeping with this finding and with those observed with *A. muciniphila* in experimental models (145–147), a study recruiting 28 patients with T2D (14 on metformin) and 84 matched controls confirmed an association between glucose tolerance and metformin-modulated gut microbiota. In details, in addition to *A. muciniphila*, other gut microbes were more abundant in individuals receiving metformin, namely *Butyrivibrio, Bifidobacterium bifidum*, and *Megasphaera*, which are known to produce short-chain fatty acids. Conversely, *Clostridiaceae 02d06* were more abundant in patients with T2D not taking metformin (150).

In an exploratory, unblinded study (NCT01357876), Napolitano et al. recruited 12 T2D patients who were receiving a stable dose of metformin (≥1,000 mg/day) for more than 3 months. They collected post-prandial blood samples, stool samples, upper small intestine bile, and fasting plasma samples for metformin concentrations at scheduled intervals. They observed that abundance of the *Firmicutes* in the microbiota was positively correlated with changes in cholic acid and conjugates, while *Bacteroidetes* abundance was negatively correlated with those of bile acids. Both were also correlated with levels of serum peptide tyrosine–tyrosine, further highlighting a complex gut-based pharmacology underlying the mechanism of action of metformin (152).

Indeed, modulating the gut microbiota by increasing favorable phyla, such as *Akkermansia* spp., might enhance the antidiabetic effects exerted by metformin. On the other side, microbiota modifications induced by metformin may affect immune function. In NOD mice, *A. muciniphila* has been demonstrated to protect from the development of islet cell autoimmunity (153) and restore intestinal immunity and homeostasis in experimental models of IBD (154, 155).

The relationship between metformin and microbiota is likely bi-directional, with a pronounced microbial effect on the mechanisms of action and efficacy of the drug, and with metformin affecting functions and abundance of specific phyla. Further data on this complex drug–microbiota interplay may extend our knowledge on metformin and identify novel targets for tailored treatments based on gut microbiota manipulation exploitable in autoimmune diseases.

## Conclusion

Metformin is a safe, inexpensive medication with a history of more than 50 years of clinical experience in treating patients with T2D. In the last decade, several preclinical and clinical studies highlighted pleiotropic beneficial effects of this molecule on other clinical domains, including cancer susceptibility and cardiovascular disease risk. More recently, different *in vitro* studies demonstrated that metformin can regulate the function of many cell types involved in autoimmunity development and maintenance. Concurrently, metformin has been shown to be able to restore immune homeostasis and improve disease severity in animal models of autoimmune diseases. Based on this preclinical background and because of a well-established safety profile, metformin should be reconsidered in clinical trials designed to prove its efficacy in patients with autoimmune diseases. Largely available retrospective data (i.e., by analyzing RA patients treated with metformin for comorbid T2D) may contribute to provide further support to longitudinal studies. We are now going to test this hypothesis in humans through a double-blind placebo-controlled clinical trial (Metformin Treatment in Systemic Sclerosis, METSS—EudraCT number: 2018-000733-12) that recently received funding from Italian Ministry of Health. Time will tell us whether a well known and relatively safe drug, will be a new “bullet” available to physicians dealing with patients with autoimmune/rheumatological conditions.

## Author Contributions

FU, ER, and RD conceived the review idea. FU, ER, GP, SD, AC, GD, RM, and RD contributed to prepare the first draft of the manuscript. GD, RM, and RD critically revised the final draft. All the authors discussed the results and contributed to the final manuscript.

## Conflict of Interest Statement

The authors declare that the research was conducted in the absence of any commercial or financial relationships that could be construed as a potential conflict of interest.
